# Inflammatory mediators in nasal lavage among school-age children from urban and rural areas in São Paulo, Brazil

**DOI:** 10.1590/S1516-31802004000500005

**Published:** 2004-09-01

**Authors:** Clóvis Eduardo Santos Galvão, Paulo Hilário Nascimento Saldiva, Jorge Elias Kalil, Fábio Fernandes Morato Castro

**Keywords:** Nasal lavage fluid, Hypersensitivity, Allergens, Inflammation mediators, Inflammation, Liquido da lavagem nasal, Alérgenos, Mediadores da inflamação, Inflamação, Hipersensibilidade

## Abstract

**CONTEXT:**

Some studies have shown that inflammatory processes in the nasal air passages may reflect or affect those in the lower airways. We decided to indirectly assess the inflammatory status of the nasal airways in two groups of children with different sensitization rates to aeroallergens.

**OBJECTIVE:**

To compare the inflammatory activity in the nasal airways, through the determination of mediators in nasal lavage fluid in two distinct populations.

**TYPE OF STUDY:**

Cross-sectional study.

**SETTING:**

Two public elementary schools, one in an urban setting and the other in a rural setting of the State of São Paulo, Brazil.

**METHODS:**

Two groups of 40 elementary school children with different sensitization rates to aeroallergens were formed. Samples of nasal lavage fluid were assessed for eosinophil cationic protein (ECP) and tryptase. Non-parametric tests were used for statistical analysis.

**RESULTS:**

Significantly higher levels of ECP were observed among students living in the urban area than those in the rural area (p < 0.05). No significant difference in the tryptase levels was observed. Also, the urban children who were sensitized to aeroallergens presented higher levels of ECP in nasal mucosa than the non-sensitized children, while this difference was not observed among the rural children.

**DISCUSSION:**

The lack of mast cell activity and increased eosinophil degranulation revealed a chronic inflammatory state in the nasal air passages. The higher eosinophil activity in the urban area, coinciding with higher sensitization to aeroallergens, suggests that there must be some factors in the urban area that can modulate airway inflammation by influencing the activation of inflammatory cells.

**CONCLUSION:**

Our findings showed that there was no difference in the concentrations of tryptase in nasal lavage fluids between the two studied groups. However, the children from the urban area presented with higher concentrations of eosinophil cationic protein than did those from the rural area. Also, the urban children who were sensitized to aeroallergens presented with greater concentrations of eosinophil cationic protein in nasal mucosa than the non-sensitized children, while this difference was not observed among the rural children.

## RESUMO

**CONTEXTO:**

Alguns estudos demonstram que o processo inflamatório nas vias aéreas nasais poderia refletir ou mesmo afetar as vias aéreas inferiores. Decidimos avaliar indiretamente o estado inflamatório das vias aéreas nasais de dois grupos de escolares com diferente sensibilização aos aeroalérgenos mais comuns.

**OBJETIVO:**

Comparar a atividade inflamatória nas vias aéreas nasais, através da deter-minação de mediadores inflamatórios no lavado nasal em duas populações distintas de crianças em idade escolar.

**TIPO DE ESTUDO:**

Estudo transversal.

**LOCAL:**

O estudo foi realizado em duas escolas públicas de ensino fundamental, uma em zona urbana e outra em zona rural, no Estado de São Paulo.

**MÉTODOS:**

Foram constituídos dois grupos de 40 escolares que apresentam diferentes taxas de sensibilização a aeroalérgenos comuns. Amostras do lavado nasal foram colhidas para determinação de proteína catiônica eosinofílica (ECP) e triptase. Testes não-paramétricos foram usados na análise estatística.

**RESULTADOS:**

Níveis significativamente maiores de proteína catiônica eosinofílica foram encontrados nos estudantes da área urbana (p < 0,05). Não houve diferença estatística nos níveis de triptase entre os dois grupos. Observou-se ainda que, na área urbana, as crianças sensibilizadas aos aeroalérgenos apresentaram maiores concentrações de proteína catiônica eosinofílica, o que não foi observado nas crianças da zona rural.

**DISCUSSÃO:**

A ausência de atividade de mastócitos e a degranulação aumentada de eosinófilos revelaram uma inflamação crônica nas vias aéreas das crianças estudadas. A maior atividade de eosinófilos na zona urbana, coincidindo com a maior sensibilização aos aeroalérgenos, sugere que deve haver algum fator a mais na área urbana que modula a resposta das vias aéreas influenciando a ativação das células inflamatórias locais.

**CONCLUSÃO:**

Nossos achados não mostraram diferenças nos níveis de triptase no lavado nasal entre os dois grupos estudados. Por outro lado, as crianças da area urbana apresentaram maiores concentrações de proteína catiônica eosinofílica do que aquelas da zona rural. Observamos ainda que, na area urbana, as crianças sensibilizadas por aeroalérgenos apresentaram maiores concentrações de proteína catiônica eosinofílica do que aquelas não sensibilizadas, enquanto esta diferença não foi observada nas crianças da area rural.

## INTRODUCTION

Epidemiological studies have demonstrated that the incidence of respiratory allergies has been increasing over the last few decades. Most of these studies support the theory that various environmental changes could be responsible for this increase.^1,2^ Some reports suggest a higher prevalence in urban communities,^3^ but this is not confirmed by others.^4^ Ishizaki et al. (1987) compared the prevalence of pollinosis due to cedar pollen in two different areas in Japan. They reported a significantly greater prevalence in areas with high allergen and automobile exhaust exposure than in mountainous regions, thereby showing that pollution could be a critical factor in developing sensitization.^5^

Inflammation is believed to be the major event in the pathogenesis of respiratory allergy, with evidence that inflammatory mediators produced by epithelium play an important role in this process. Several reports have described relatively high expression of these mediators in the airway mucosa of allergic and asthmatic subjects.^6^

We have recently studied two groups of 40 school-aged children from rural and urban areas in the State of São Paulo, Brazil.^7^ Although the studied groups were homogenous in relation to the personal and family history of allergies, a significantly higher sensitization rate was observed among the children living in the urban area than among those in the rural area. As a continuation of this study, and on the basis of some reports showing that the inflammatory processes in the nasal air passages may reflect or affect those in the lower airways,^8^ we decided to assess the concentrations of tryptase and eosinophil cationic protein (ECP) in nasal lavage fluids in these groups of children to compare the activity of mast cells and eosinophils, respectively. Since these two mediators are known to be markers for inflammation, their concentrations in nasal lavage fluids could be related to mucosal inflammation.

## METHODS

A cross-sectional study was performed using groups of students recruited from two public elementary schools: Escola Municipal de Ensino Fundamental João Carlos da Silva Borges, located in Moema, City of São Paulo; and Escola Estadual de Primeiro Grau Professora Zilah Barreto Pacitti, in the Portão district, within the rural zone of the municipality of Atibaia, State of São Paulo. Forty students from each school took part in this study. They agreed to participate and their parents gave informed consent. The ethical committee of Faculdade de Medicina da Universidade de São Paulo approved the protocol for the study.

The assessment of these children prior to the present study had included anamneses concerning their respiratory symptoms within the last 12 months (nasal blockage, rhinorrhea, sneezing, nasal pruritus, dyspnea, wheezing, cough, snoring and ocular pruritus), their personal and family history of atopy, a battery of skin prick tests using a panel of common aeroallergens and the children's use of medication, as described elsewhere.^7^ The children underwent a single nasal lavage procedure, provided that they had been asymptomatic for 15 days prior to the investigator's visit to the school, and provided that no use of anti-inflammatory medications or anti-histamines had been made for at least 30 days. A modification of the technique in Hilding, 1972,^9^ as described by Wang et al.^1^ in 1995, was used.

Two of the children at the São Paulo school were symptomatic and using medications at the time of the visit. Also, two students from São Paulo and one from Atibaia had moved to different schools. Thus, the nasal lavage was carried out on 36 children from São Paulo, and 39 from Atibaia.

A catheter of size 12 Foley (Embramac, Euromedical, Malaysia) was placed in the nasal vestibule. The balloon was inflated to the maximum comfortably tolerated by the child, so as to create a seal. Each subject was asked to flex his or her head slightly forward to prevent escape of fluid, and lavage was performed by connecting the catheter to a syringe containing 7 ml of normal saline. The cycle of lavage and aspiration was performed three times. The balloon was then deflated and the catheter removed. Samples of the nasal lavage fluids were stored at –70° C until analysis was performed. The median return of the total input of saline solution was 50% for the São Paulo subjects (range: 22% to 86%) and 42% for the Atibaia subjects (range: 23% to 68%). The concentration of ECP and tryptase were measured by an antibody fluoroenzyme immunoassay method (UniCAP Pharmacia & Upjohn, Uppsala, Sweden). The detection limits were 2-200 μg/l for ECP and 1-200 μg/l for tryptase.

Non-parametric tests (Fisher exact test and Mann-Whitney U-test) were used for statistical analysis. The Kolmogorov-Smirnov test was applied in the analysis of ECP and tryptase levels, to check whether the distribution of pairs of samples was homogeneous, so that they could be compared. The values of ECP were logarithmically transformed because they were not normally distributed. The analyses were performed using Statistica 5.0 for Windows^®^ and the Statistical Package for the Social Sciences (SPSS) 10.0 for Windows^®^. Statistical significance was defined as p < 0.05.

## RESULTS

The ages of the students participating in this study ranged from 9 to 16 years in São Paulo, and from 12 to 18 years in Atibaia, with predominance of females (52.5%) in São Paulo and males (65%) in Atibaia. The two groups of children were similar in terms of personal and family history of allergy and respiratory symptoms during the last 12 months, as well as their prior use of medication, as reported elsewhere.^7^ Skin prick tests were positive for at least one of the allergens in 19 subjects (47.5%) in São Paulo and in 10 subjects (25%) in Atibaia, and this difference was statistically significant (p < 0.05) (Table 1).

**Table 1:** Variables studied in 80 school children from Atibaia and São Paulo

Variable	Atibaia (n = 40)	São Paulo (n = 40)	p-values
Personal history of allergy	11 (27.5%)	14 (35%)	p = 0.315
Family history of allergy	10 (25%)	9 (22.55%)	p = 0.500
Passive smoker	22 (55%)	27 (67.5%)	p = 0.179
Indoor plants	37 (92.5%)	36 (90%)	p = 0.500
Contact with animals	37 (92.5%)	26 (65%)	p = 0.003
Positive skin prick test	10 (25%)	19 (47.5%)	p = 0.031

There was no statistically significant difference in the levels of tryptase between the groups, in the nasal lavage fluids (Table 2). The levels of ECP were significantly higher in São Paulo than in Atibaia (p < 0.05). It was also observed that in São Paulo, the ECP levels were significantly higher in the nasal lavage fluids of sensitized children, in comparison with those with negative skin prick tests, while this was not observed in Atibaia (Table 3).

**Table 2:** Inflammatory mediators in nasal lavage fluids in 75 school children from Atibaia and São Paulo

Variable	Atibaia	São Paulo	p-values
ECP (ln)	n = 39	n = 36	
μ ± sd	1.75 ± 1.55	2.08 ± 1.46	p = 0.048
Median	0.69	1.55	
Min – Max	0.69 – 5.30	0.69 – 5.30	
Tryptase	n = 39	n = 36	p = 0.959
μ ± sd	0.017 ± 0.087	0.036 ± 0.19	
Median	0.00	0,00	
Min – Max	0.00 – 0.54	0.00 – 1.15	

*μ = mean; sd = standard deviation; ln = logarithm; ECP = eosinophil cationic protein.*

**Table 3:** Correlation between prick test and eosinophil cationic protein levels in nasal lavage fluids in 75 school children from urban (São Paulo) and rural (Atibaia) areas

		Atibaia	São Paulo	p-values
**ECP Levels**	**Positive prick test**	n = 10	n = 18	p = 0.774
	μ ± sd	2.53 ± 2.07	2.46 ± 1.38	
	Median	1.69	2.21		
	Min – Max	0.69 – 5.30	0.69 – 5.00	
	**Negative prick test**	n = 29	n = 18	p = 0678
	μ ± sd	1.48 ± 1.26	1.71 ± 1.47	
	Median	0.69	0.79		
	Min - Max	0.69 – 4.44	0.69 – 5.30		
	**p-values**	p = 0.18	p = 0.041	

*μ = mean; sd = standard deviation; ns = non-significant; ln = logarithm; ECP = eosinophil cationic protein.*

Univariate and multiple regression models were used to verify the variables that were consistently associated with positivity in skin prick tests. These models showed that the group living in São Paulo had a high risk of sensitization [odds ratio (OR) = 3.96; 95% confidence interval (CI) = 1.22-12.84], as did those with higher levels of ECP in the nasal lavage fluids (OR = 1.44; 95% CI = 1.01-2.06).

## DISCUSSION

Our findings showed that there was no difference in the concentrations of tryptase in nasal lavage fluids between the two studied groups.

However, the children from São Paulo, the urban area, presented with higher concentrations of ECP than those from Atibaia. It was also observed that in São Paulo, the children sensitized to aeroallergens presented with greater concentrations of ECP in nasal mucosa than the non-sensitized ones, while this difference was not observed in children from Atibaia.

To continue the study^7^ of the two groups of school-aged children from urban and rural areas in the State of São Paulo, Brazil, we decided to assess the nasal inflammatory status of these students. Nasal inflammation may have relevance to lower airway inflammatory disease such as asthma and also may provide indirect information about bronchial inflammation.^10^ Moreover, because the procedure required for obtaining nasal secretions is relatively less invasive than for bronchial specimens, we assessed nasal lavage fluid to analyze the inflammatory response in the nasal mucosa due to the inflammatory mediators from mast cells and eosinophils.

We focused on tryptase and ECP determinations to assess the nasal mucosa inflammation, since increases in these two components in body fluids have been considered to be markers for mast cell and eosinophils activation.^10,11^ Thus, we could make an assessment of acute and chronic inflammation, respectively.

Some studies have been performed by comparing the concentrations of inflammatory mediators in nasal lavage fluids before and after experimental nasal allergen challenge, but this has not been done in relation to natural allergen exposure. Furthermore, few detailed quantitative data on normal and pathological concentrations regarding these mediators in nasal secretions have been published up to this date.

It is known that mast cells play an important role in allergic inflammation. Tryptase is the major neutral protease in human mast cells.^10^ We observed no signifi-cant levels of tryptase in nasal lavage fluids, either in São Paulo or in Atibaia. This was already expected, since we were assessing this mediator under “baseline” conditions (not allergen-challenged and not during exacerbation of symptoms). It has also been previously reported that natural allergen exposure did not increase tryptase levels in nasal lavage fluids.^12^ In contrast, the concentrations of ECP, a granule-derived protein released upon eosinophil degranulation, were significantly higher in the São Paulo group than among those from Atibaia. In 1995, Noah et al.^13^ demonstrated higher levels of ECP in the nasal lavage fluids from asthmatics, in comparison with normal subjects. Some authors have reported modifications of inflammation markers in nasal lavage samples during and outside of the pollen season.^14^ These authors observed that, during the pollen season, ECP and tryptase levels increased in nasal lavage, and that tryptase correlated with the symptoms. In the present study, there was no correlation between the levels of ECP and respiratory symptoms, since we performed the nasal lavage procedure only on asymptomatic students. There was also no correlation between the levels of ECP and tryptase and the personal history of respiratory allergy.

We hypothesized that sensitized children would have increased concentrations of inflammatory mediators in nasal lavage fluids in both groups. However, when we compared the data, we found out that in São Paulo, sensitized subjects had higher levels of ECP than non-sensitized children, while this was not observed in Atibaia. This finding shows that sensitization is not enough to explain higher concentrations of ECP in nasal mucosa of some of the children. The activation of eosinophils is not completely understood, but it is believed that non-specific agents could degranulate these cells, thus leading to chronic inflammatory status in nasal airways. It can therefore be speculated that non-specific or irritant agents present in the urban environment could be responsible for the higher ECP concentrations in nasal lavage fluids. The so-called western lifestyle, represented by modifications in housing conditions (such as the use of carpets and air conditioners), modifications in dietary habits, smoking, stress and air pollution, has been mentioned as the possible cause of continuous irritation to the airway mucosa.^2^ We are about to finish another study in which we analyze the degree of exposure to aeroallergens (dust mites and molds) in each group. Soon, we will be able to answer whether there is any difference in allergen content between these groups. However, further studies will be necessary to better explain the difference in sensitization between these children.

Despite some controversy, several studies have already reported higher incidence of respiratory allergies in polluted cities, such as the study by Braback et al.^15^ That study reported on comparisons between urban and rural children in Sweden and Poland, showing that urban children from both countries had higher prevalence of sensitization. Some other studies have shown that skin prick test positivity is higher among children living in urban areas.^16^ The exposure to air pollutants is significantly different between the two populations in the present study. São Paulo is heavily polluted, while in Atibaia the air pollution is considered negligible.^17^ Thus, the next step in this study would be a quantitative analysis of pollutants to evaluate the role of air pollution in the sensitization of these children.

## CONCLUSION

The finding of no mast cell activity and increased eosinophil degranulation in nasal lavage fluids revealed a chronic inflammatory state in the nasal air passages. The higher sensitization to aeroallergens in São Paulo, coinciding with the higher eosinophil activity, especially among the sensitized children, suggests that there must be some factors in São Paulo that can modulate airway inflammation by influencing the recruitment and activation of inflammatory cells. Further studies on the levels of exposure to allergens and pollution in these two populations would help in better understanding the differences in sensitization rates.
